# Circular Metagenome-Assembled Genome of *Methanobacterium* sp. Strain ERen5, a Putative Methanogenic, H_2_-Utilizing Terrestrial Subsurface Archaeon

**DOI:** 10.1128/mra.00676-22

**Published:** 2022-09-06

**Authors:** Daniel Lipus, Zeyu Jia, Alexander Bartholomäus, Oliver Burckhardt, Megan Sondermann, Dirk Wagner, Jens Kallmeyer

**Affiliations:** a GFZ German Research Centre for Geosciences, Section Geomicrobiology, Potsdam, Germany; b University of Potsdam, Institute of Geoscience, Potsdam, Germany; Portland State University

## Abstract

A circular, single-contig *Methanobacterium* sp. metagenome-assembled genome (MAG) was recovered from high-CO_2_ enrichments inoculated with drill core material from the tectonic Eger Rift terrestrial subsurface. Annotation of the recovered MAG highlighted putative methanogenesis genes, providing valuable information on archaeal activity in the deep biosphere.

## ANNOUNCEMENT

With frequent seismic activity and consistently high CO_2_ fluxes, the Eger Rift in Western Bohemia (1) represents a rare subsurface ecosystem and a scientifically relevant location to study microbial behavior and biological-geological interactions in the deep subsurface (2–4). Seismic activity in this region has been suggested to release H_2_, thereby promoting the production of biogenic methane through methanogenic *Archaea* (5).

Retrieval of sediment and rock samples from a 240-m core drilled at the Hartusov mofette field (Czech Republic) allowed the enrichment of native microbial communities. Drill core materials (~5 g each) from eight different depths (46 m to 230 m) were separately enriched in slurries using OMV5 mineral medium under CO_2_/H_2_ headspace and incubated at 16°C for 3 months. For the recovery of the here-reported draft genome, genomic DNA was extracted from the enrichment slurry of 54-m-deep, mudstone composited drill core material, using the FastDNA isolation kit (MP Bio, Irvine, CA, USA). High-molecular-weight DNA was prepared using the rapid barcoding sequencing kit (Oxford Nanopore Technologies [ONT], Oxford, UK) and cleaned up using AMPure XP beads (Beckman Coulter, Pasadena, CA), removing small DNA fragments. The resulting library was sequenced using the MinION platform (ONT) and the Flo-MIN106 flow cell for 72 h. Sequencing raw data was basecalled and demultiplexed using high accuracy with guppy v4.4.2 + 9623c1626 (ONT) resulting in 398,466 raw reads with an *N*_50_ of 3852bp. Default parameters were used for all software unless otherwise specified. Assembly and polishing were performed with Flye v2.8.2-b1689 (6) (parameters: –plasmid –meta). Assembly produced (among others) a single, 2,744,370-bp-long contig, which Flye specified to be circular. Binning via MetaBAT2 (7) confirmed this contig to represent a metagenome-assembled genome (MAG). MAG quality was assessed using the lineage_wf workflow, and full-length 16S rRNA sequences were recovered using the ssu_finder tool of Check Mv1.0.13 (8).

The recovered MAG was found to have an average coverage of 364 and a GC content of 35.5% and was estimated to be 98.0% complete and 0.0% contaminated. Taxonomic assessment using GTDB-TK v1.5.0 (9) classified the recovered MAG as *Methanobacterium*. The draft genome was compared to other *Methanobacterium* genomes by calculating the average nucleotide identity (ANI) using the JSpeciesWS tool (accessed March 2022) (10). *Methanobacterium* sp. strain ERen5 shares the most nucleotide-level genomic similarity with Methanobacterium lacus strain AL-21 (ANIb = 91.45% and ANIm = 92.56%). Full-length 16S rRNA sequence comparison (1,488 bp) using NCBI BLAST (11) revealed 99.5% sequence identity to Methanobacterium lacus strain 17A1, isolated from the profundal sediments of a freshwater meromictic lake (12).

Annotation using PGAP (13) allowed the identification of putative hydrogenotrophic, methanogenesis genes, including coenzyme B sulfoethylthiotransferase alpha subunit *mcrA* (locus tag NKF70_05265, EC 2.8.4.1) and N5-methyltetrahydromethanopterin:coenzyme M methyltransferase subunit *mtrA* (locus tag NKF70_01070, EC 2.1.1.86). Phylogenetic assessment of *mcrA* suggested the recovered genome to be closely related to Methanobacterium lacus and Methanobacterium paludis strains isolated from northern peatlands (14).

### Data availability.

The draft genome of *Methanobacterium* sp. strain ERen5 was deposited at NCBI with the accession number CP099988 under BioProject PRJNA832091. Raw reads are accessible via the accession number SRR19049537. The 16S rRNA sequence is available at NCBI under the accession number ON341022.1.
